# 

**Published:** 1789

**Authors:** 


### CONTENTS.

Page

I. *AN Account of an epidemic Sore Throat which appeared at Chesham, in Buckinghamshire, in the Year* 1788. *By Mr.* Henry Rumsey, *jun. Surgeon at Chesham, and Member of the Corporation of Surgeons of London.* - - - - - - 7

II. *A Case of Cancer of the Breast; with Remarks. By Mr.* T. Hughes, *Surgeon at Stroud-Water, in Gloucestershire.* - - 40

III. *A Case of Hernia. By Mr.* Thomas Clowes, *Member of the Corporation of Surgeons of London, and Surgeon at Wingham, in Kent.* - - - - - - 72

IV. *A Case of Tæniæ Hydatigenæ, or Hydatids, successfully treated by the Use of Mercury. By* James Lind, *M. D. F. R. S. Physician at Windsor, Fellow of the Royal College of Physicians and of the Royal Society of Edinburgh.* - - - - - - - 76

V. *An Account of the successful Application of the Trepan in a Disease of the Tibia. By M.* Verguin, *Surgeon Major of the Naval*

*Hospital*

Page

*Hospital and Inspector of the Royal College of Surgery at Toulon. From the Journal de Medecine Militaire,* Tome VII. - - 80

VI. *A remarkable Case of numerous Births; with Observations. By* Maxwell Garthshore, *M. D. F. R. S. and A. S. in a Letter to Sir* Joseph Banks, *Bart. P. R. S. From the Philosophical Transactions of the Royal Society of London, Vol.* LXXVII. *for the Year* 1787. *Part* II. - - - - - - 83

Catalogue *of* Books. - - - 99

